# Research Contributing to Improvements in Controlling Florida’s Mosquitoes and Mosquito-Borne Diseases

**DOI:** 10.3390/insects7040050

**Published:** 2016-09-28

**Authors:** Walter J. Tabachnick

**Affiliations:** Florida Medical Entomology Laboratory, University of Florida, IFAS, 200 9th St. SE, Vero Beach, FL 32962, USA

**Keywords:** mosquito control, Rotational Impoundment Management, adulticiding, arbovirus surveillance

## Abstract

Research on mosquitoes and mosquito-borne diseases has contributed to improvements in providing effective, efficient, and environmentally proper mosquito control. Florida has benefitted from several research accomplishments that have increased the state’s mosquito control capabilities. Research with Florida’s mosquitoes has resulted in the development of ecologically sound management of mosquito impoundments on Florida’s east coast. This strategy, called Rotational Impoundment Management (RIM), has improved the ability to target the delivery of pesticides and has helped to reduce non-target effects and environmental damage. Research has led to the development of an arbovirus surveillance system which includes sentinel chicken surveillance, real time use of environmental contributing factors like meteorology and hydrology to target mosquito control, as well as public health efforts to mitigate disease outbreaks to areas with risk of disease. These research driven improvements have provided substantial benefits to all of Florida. More research is needed to meet the future challenges to reduce emerging pathogens like Zika virus and the consequences of environmental changes like global climate change that are likely to influence the effects of mosquito-borne pathogens on human health and well-being.

## 1. Introduction

Research on mosquitoes and humankind’s war against mosquito pests and mosquito vectors entered the modern era at the beginning of the 20th century with the U.S. Yellow Fever Commission’s demonstration that *Aedes aegypti* transmitted the dreaded yellow fever virus (YFV) [1]. This subsequently led to the first triumphs against mosquito-borne disease through effective mosquito control spearheaded by William Gorgas who directed the successful control of *Ae. aegypti* in Havana Cuba in 1900, and then again in the Panama Canal construction project completed in 1914. As a result, yellow fever was virtually eliminated from Havana in 1902 for the first time in approximately 300 years, and later essentially eliminated in the Panama Canal Zone, which contributed greatly to the successful completion of the canal by the U.S. Previously, France had failed in its attempt to build a canal across the Isthmus of Panama nearly 25 years before in part due to malaria and yellow fever epidemics. Since that time much knowledge has been accumulated about mosquito biology for many of the approximately 3500 described species of mosquitoes throughout the world, with greatest attention to the species of mosquitoes that are the major human pests and those that transmit a variety of pathogens affecting human and non-human hosts. General information on mosquito biology and mosquito control can be found elsewhere [2,3,4].

Knowledge of mosquitoes has been vital to humankind’s ability to control mosquitoes and mosquito-borne diseases. The myriad of ways such knowledge has improved mosquito control are beyond the scope of this review. An introduction to the vast biological literature is available elsewhere in selected reviews describing the influence on mosquito control of systematics [5], population genetics [6], genetics [7], behavior [8], physiology, and ecology [9]. Research in California and collaborations with California mosquito control programs reviewed elsewhere [10] continue to improve mosquito control [11].

Here I outline the impact of research on mosquito control methods by discussing studies in Florida and the improvements these efforts have had on mosquito control. Florida’s habitats and the resulting large mosquito populations were the reasons for organizing Florida’s first mosquito control district nearly 100 years ago. There are now mosquito control organizations in many parts of the state to fight what are considered nuisance or pest mosquitoes, and to also combat Florida’s species of mosquitoes that transmit mosquito-borne pathogens [12,13]. The mission of Florida’s mosquito control organizations is to protect residents and visitors from mosquitoes using effective, efficient, and environmentally proper mosquito control [14].

Florida’s mosquito control professionals, represented by the Florida Mosquito Control Association, believe that information about mosquito biology is essential to accomplish its mission to protect the public from mosquitoes [14]. Studies in Florida have provided information about disease surveillance, mosquito biology, wetlands ecology, human-made mosquito problems, disease prevention, repellents and attractants, improving existing pesticide application technology, resistance to insecticides, non-target organisms, and biocontrol [15]. One study on sampling mosquitoes and the factors influencing mosquito flight patterns illustrates the long history of Florida’s research on mosquitoes and mosquito control [16].The commitment to obtain knowledge about mosquito biology in Florida led to the development of a Florida state-supported mosquito biology research program to improve mosquito control more than 60 years ago under the leadership of John H. Mulrennan Sr. and Dr. Maurice Provost, both then with the Bureau of Entomology of the Florida State Board of Health [13]. Their leadership resulted in the creation of the Florida Entomological Research Center that later became the University of Florida’s Florida Medical Entomology Laboratory (FMEL) in Vero Beach. This later led to the John Mulrennan Public Health and Entomology Research and Education Center (PHEREC) when several of the Vero Beach faculty were relocated to Panama City. PHEREC later became part of Florida A & M University until it was closed in 2011. Much of the information on the biology of Florida’s mosquitoes and the use of this information to improve mosquito control were due to the efforts of the faculty and staff at these two laboratories working in collaboration with many of Florida’s mosquito control and public health professionals. Table 1 shows the extent of some of the contributions of these two laboratories since 1960 to several selected subjects about the biology of Florida’s mosquitoes and several of the pathogens they transmit. From 30%–60% of all peer reviewed articles involving Florida mosquito ecology, Florida *Aedes aegypti*, Florida mosquito control, Florida St. Louis encephalitis, and Florida West Nile virus were produced by these laboratories. The efforts of the laboratories have been supported over the years by the Florida Mosquito Control Research Program (FMCRP). This program is administered by the Florida Department of Agriculture and Consumer Services (FDACS) that is legislatively authorized to use not more than 20% (Florida Statute 388.261) of Florida’s funds from taxes generated by the Florida waste tire tax to support the research program (Florida Statute 403.709). Florida mosquito control efforts have also benefitted from research conducted by the U. S. Department of Agriculture (USDA), Agricultural Research Service, Center for Medical, Agricultural and Veterinary Entomology (CMAVE), located in Gainesville, Florida. Though CMAVE’s mission addresses national issues, CMAVE scientists have provided a substantial amount of information on trapping, surveillance, and biocontrol that has also been valuable to Florida mosquito control [15].

The goals of this manuscript are to: (1) review three research areas resulting from programs supported in part by the FMCRP, and/or provided by FMEL or PHEREC, and/or conducted by Florida’s mosquito control organizations: Rotational Impoundment Management (RIM), mosquito spray technologies and non-target effects of mosquito control, and arbovirus surveillance; (2) provide information on some of the impacts of these accomplishments in improving mosquito and mosquito-borne disease control; (3) present examples of current challenges requiring research on the biology of mosquitoes.

## 2. The Florida Mosquito Control Research Program (FMCRP)

The FMCRP has provided state funds for research to improve mosquito control and the ability to effectively combat mosquito-borne diseases throughout Florida for nearly 30 years [17]. The program is managed by FDACS currently with an annual budget of $500,000. These funds support projects that are important to the entire state. For example, information was provided through the FMCRP on the biology of the primary mosquito vectors of West Nile virus (WNV), St. Louis encephalitis virus (SLEV), and eastern equine encephalitis virus (EEEV) in Florida. This information is the basis of Florida’s ability to reduce populations of the dangerous vector mosquitoes reviewed below. Information provided by FMCRP supported projects on new insecticides, novel strategies to mitigate mosquito populations while reducing the impact of mosquito control practices on the environment, and on non-target organisms and new biocontrol methods. The FMCRP provided the information that is the basis of Florida’s sentinel chicken surveillance system to assess transmission of several arboviruses and serve as an early warning system for epidemics. This system has been Florida’s first line of defense against mosquito-borne disease like West Nile fever, and St. Louis and eastern equine encephalitides. The Florida Coordinating Council on Mosquito Control (FCCMC) consists of representatives of several Florida state agencies including Florida’s Department of Agriculture and Consumer Services (FDACS), Department of Health, Department of Environmental Protection, Fish and Wildlife, several environmental groups, USDA, Florida Mosquito Control, and Florida’s state research universities. Each year the FCCMC recommends the high priority needs for information to improve mosquito control to FDACS. The established priorities are then used by an independent research selection committee of scientific experts administered by FDACS. This committee evaluates the quality of submitted projects to ensure that selected projects provide important, high priority information while also being scientifically sound.

The FMCRP goal is to provide information that will directly benefit Florida mosquito and mosquito-borne disease control. The focus of this paper is on research studies in Florida, in large part supported by the FMCRP, to the exclusion of many important research studies elsewhere that have also benefitted mosquito control. The references provided here will lead readers to the extensive literature describing other contributions. The following are a few selected accomplishments that illustrate the impact of Florida’s research programs on the biology of mosquitoes and on methods to control them.

## 3. Improved Mosquito Impoundments Using Rotational Impoundment Management (RIM)

**Figure 1:**
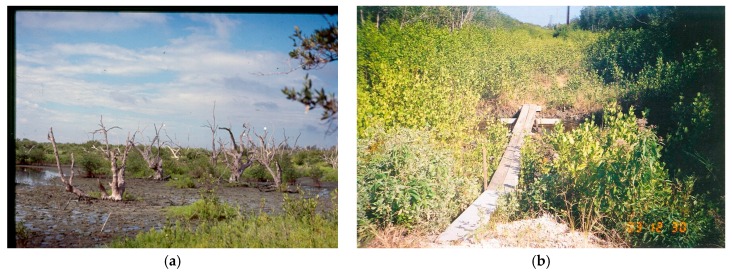
(**a**) A mosquito impoundment not connected to the adjacent Florida Indian River Lagoon resulting in the loss of the wetland vegetation; (**b**) A mosquito impoundment reconnected to the adjacent Florida Indian River Lagoon and maintained using Rotational Impoundment Management (RIM) resulting in restoration of wetland vegetation (Courtesy of Douglas B. Carlson).

Many methods are available to help control mosquito production from wetlands [18]. For example, along Florida’s east coast many salt marsh or mangrove forest areas produce huge populations of *Aedes taeniorhynchus* and *Aedes sollicitans* that are effectively controlled using impoundments. These species of mosquitoes do not lay their eggs on standing water but prefer moist marsh soils [19]. Dikes are used to separate the impounded area from nearby waterways on Florida’s east coast in the Indian River Lagoon that are utilized to control the water levels in the impounded areas for mosquito control. Flooding the impounded areas at times of the year when saltmarsh mosquitoes are seeking moist substrate to lay eggs prevents oviposition and results in fewer mosquitoes in the next generation. Research demonstrated that impounded wetlands had many detrimental effects on the wetlands ecology [20]. The impoundments degraded wetlands, they allowed nonnative plants to invade, and they interfered with the migration and reproduction of fish and other organisms that also used the wetland habitat (Figure 1a).

Decades of research has shown that mosquito production can be halted by reconnecting impoundments to the lagoon via culverts and flooding the impoundments only during the summer mosquito re-producing season (September through May) [21,22,23]. Opening the culverts allow fishes, shrimps, and crabs to regain access to the wetland. Wetland vegetation is also restored (Figure 1b). This mosquito control method is known as RIM [20,22,24].

The benefits of the research on impoundments and RIM have been substantial. There have been significant savings to east Florida mosquito control in not having to apply costly larvicides because of RIM. RIM has extended the usefulness of mosquito impoundments to control both saltmarsh mosquitoes and improve natural resources. The savings in using RIM rather than spraying the estimated 40,000 impounded acres of marsh on the east coast of Florida is roughly $10,000,000 annually (40,000 × $25/acre for *Bti* × 10 treatments annually) [25]. RIM has returned 40,000 acres in east Florida to productive marsh acreage, maintaining their economic value to the area that has been estimated at $10,000/acre annually. This represents an economic benefit of approximately $400,000,000 annually.

## 4. Improved Pesticide Application

**Figure 2:**
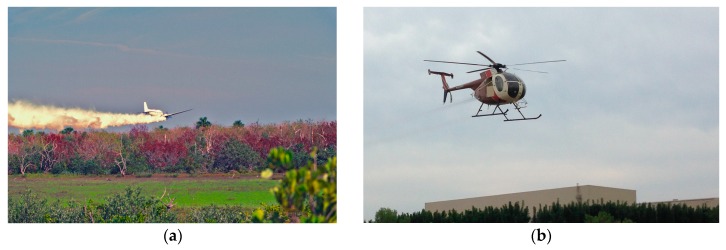
(**a**) An aerial adulticiding mission using fogging (Courtesy of G. Alan Curtis); (**b**) An aerial adulticiding mission using ultra-low volume (ULV) (Courtesy of Mark D. Latham).

Adulticides and larvicides, delivered aerially or by ground vehicles, are important tools for mosquito control. Ultra-low volume (ULV) sprays have been the preferred method for delivery of mosquito control adulticides for nearly 60 years (Figure 2). ULV is the minimum effective volume of the product without any further dilution [26]. The history and development of ULV applications for mosquito adulticides are reviewed in more detail elsewhere [27,28,29].

Research has greatly improved the effectiveness, efficiency, and environmental propriety of adulticiding for mosquito control [26,29,30]. Studies provided information on the importance of the adulticide droplet size on the effectiveness of aerial ULV [27,31,32], the calibration of spray, dosage rates and effects of different spray systems on droplet size spectrums [33,34,35,36,37], the importance of meteorological effects on adulticide applications [27,35,38,39,40,41], barriers to adulticide effectiveness [26,42,43,44], improvements to measure pesticide residues to reduce non-target effects [45], and tests to gauge the non-target effects of mosquito control ULV pesticides [46,47,48,49,50].

**Figure 3:**
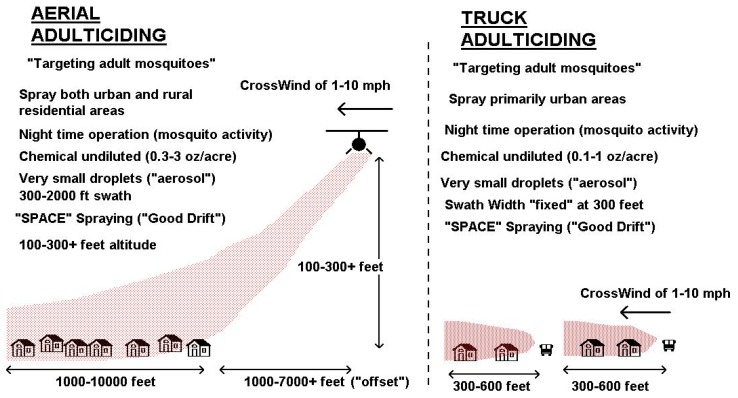
Criteria for successful aerial and vehicle ULV adulticiding (Courtesy of Mark D. Latham).

The benefits of research on ULV applications has provided Florida mosquito control organizations with greater precision and accuracy for adulticide applications. Latham [51] advised that the ideal aerosol for mosquito control would: (1) have a high ability to impact a mosquito; (2) remain airborne for long periods, thus increasing the probability of contacting a mosquito; (3) not contain more than a single toxic dose in a droplet to avoid wasting pesticide. The studies above showed that adulticides are most effective when meteorological conditions are favorable to enable the spray cloud to drift to the target area, avoid barriers such as vegetation and canopy, and avoid high deposition levels with effects on non-target species. Figure 3 provides some of the criteria used to ensure a successful adulticiding mission. The savings to mosquito control is due to having greater ability for adulticiding to impact mosquitoes. There is more precision to impact specific target areas with a dose that is the minimal dose of pesticide capable of effectively killing the mosquito, with spray that does not have local high deposition in an area, and therefore with minimal non-target effects of the adulticide. Targeting adulticide missions to impact areas with large numbers of mosquitoes reduces the need for more widespread adulticiding [52].

The savings due to improved adulticiding methods that have increased mosquito control efficiency, effectiveness, and environmental propriety vary depending on variations between mosquito control organizations in their reliance on mosquito adulticides. Manatee County Mosquito Control District (MCMCD) illustrates the potential impact of improved ULV adulticiding methods [52]. MCMCD estimates that converting adulticide sprays to more effective droplet sizes, approximately 10–30 microns in diameter, provides a significant improvement for reducing mosquito populations while using similar or lower application rates. Prior to changing droplet spectrums MCMCD applied the adulticide Dibrom at 0.67–0.75 oz./acre while current applications use 0.5–0.6 oz/acre or 20%–25% less material to treat the same area [52]. MCMCD treats on average approximately 750,000 acres annually with Dibrom currently at a cost of approximately $200 per gallon, resulting in a savings of approximately $190,000 a year due to using the smaller droplet size. In addition, MCMCD uses improved targeted delivery of adulticides that includes GPS and meteorology that takes advantage of wind effects for spray drift to minimize high adulticide deposits that might impact non-target organisms on the ground or in nearby waters [51]. MCMCD currently applies aerial adulticide sprays only at sunset and later to minimize impacts on day-active honeybees. Research on the impact of adulticides on honeybees showed that the newer spray systems that produced the smaller droplet sizes were far less damaging on bees clustered outside the hive and had no impact on overall honey yield [48]. This is a substantial improvement when compared to the previously used larger droplet sprays where higher mortality was seen in bees clustered on the outside of hives and honey yield was believed to have been impacted.

## 5. Florida Arbovirus Surveillance, Predicting Epidemics, and Mosquito Control

Studies in Florida have focused on the *Culex*-borne arboviruses beginning with studies on the mosquito vectors responsible for the SLEV Tampa epidemic in 1962 [53]. Ensuing outbreaks of SLEV like the 1990 south Florida epidemic, and in recent years the transmission of WNV in Florida by the same *Culex* vectors of SLEV, focused research on the conditions in Florida that increase risk for arbovirus transmission. This research has improved Florida’s capability to predict arbovirus transmission by *Culex* in Florida and is reviewed elsewhere [54,55].

Florida mosquito control and public health professionals use an arbovirus surveillance program built upon research to assess the risk for an arbovirus epidemic of the *Culex*-borne arboviruses, especially SLEV, WNV, and EEEV [56,57]. The goal is to use surveillance to take actions to prevent or mitigate an epidemic through targeted mosquito control to high-risk areas and to provide warnings to individuals in those areas to take precautions to reduce human infections.

**Figure 4:**
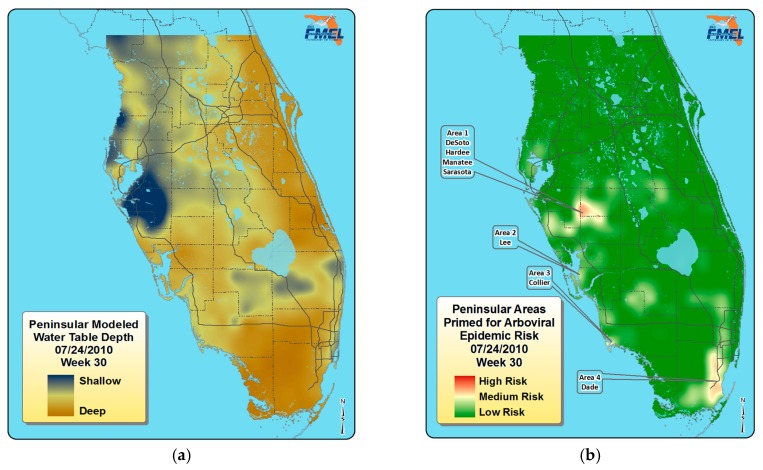
(**a**) A hydrological map for south Florida showing the ground water table during a one-week period in 2010; (**b**) A risk map for West Nile in Florida based on this hydrological information, models, and knowledge about reservoirs, vectors, and virus amplification and high-risk regions for WNV transmission (Courtesy of Jonathan F. Day).

Florida’s arbovirus surveillance for *Culex*-borne arboviruses is based on the knowledge that Florida’s mosquito vectors of these arboviruses, particularly *Culex nigripalpus* and *Culex pipiens quinquefasciatus*, primarily feed on avian hosts, have populations that are influenced by rainfall, and that substantial transmission is more likely with populations of mosquitoes with older female mosquitoes with a higher probability of having more than one or two gonotrophic cycles. Florida’s climate—which features a wide range of weather conditions, including winter freezes—also influences the risk for subsequent transmission by species of *Culex* [58,59]. Research on *Culex* vectors led to the establishment of Florida’s sentinel chicken surveillance program with hundreds of sentinel chicken flocks located throughout much of Florida. The chickens in these flocks are bled periodically (twice weekly in many districts) by mosquito control or public health professionals and sent to a testing laboratory of the Florida Department of Health to detect antibodies to EEEV, SLEV, and WNV. The results are then used to gauge the transmission in the area of the flock. *Culex nigripalpus* females have blood feeding behaviors and oviposition that are synchronized with rainfall and the flooding of temporary oviposition sites [60]. *Culex nigripalpus* and *Cx. p. quinquefasciatus* females retain their eggs until rainfall increases available oviposition sites. As a result, these females that have been infected on the first blood meal may actually complete the intrinsic incubation period during a drought, and be ready to transmit even after the first gonotrophic cycle [61]. The reliance on rainfall for large mosquito populations has led to using hydrologic conditions to predict *Culex*-arbovirus transmission in Florida [62]. The hydrological conditions in Florida shown as average depth of groundwater for a one week period (Figure 4a) and the resulting risk for WNV transmission [54] (Figure 4b) illustrate the information used to assess risk in south Florida.

**Figure 5:**
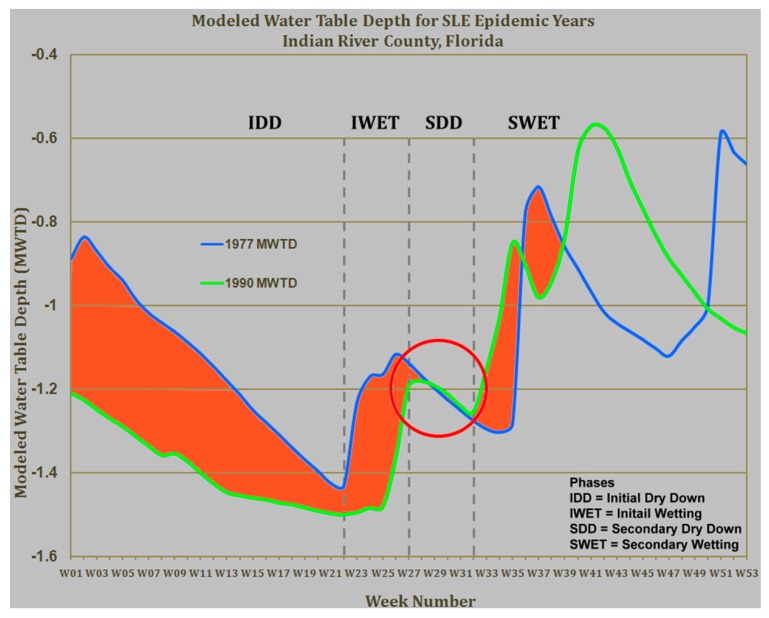
The epidemic signature of rainfall and drought events for WNV and SLEV transmission in Florida. MWTD, modeled water table depth (Courtesy of Jonathan F. Day).

Research has shown that rainfall events in Florida have a specific signature for the timing of rainfall and drought conditions that coincide with avian breeding cycles resulting in more mosquito-avian transmission that then increases the risk for concomitant human infections [62]. Specifically, spring rain events produce *Culex* populations that if followed by a period of drought will force mosquitoes and birds together around the remaining water sources. This serves to increase virus prevalence in both hosts (avians) and vectors (mosquitoes). After the drought period ends with subsequent rainfall, the *Culex* females move to the new water sources thereby increasing contact with other hosts like humans with subsequent transmission to humans. Figure 5 shows the signature for rainfall and drought events in Florida that predict *Culex*-borne epidemics of SLEV and WNV.

The age structure of *Cx. nigripalpus* or *Cx. p. quinquefasciatus* populations is another important factor in assessing risk. Samples of *Cx. nigripalpus* or *Cx. p. quinquefasciatus* females in a population can be assessed for parity, blood feeding status, and age using morphological changes in ovarian and tracheole structure that occur during egg maturation and oviposition. This can be used to establish the number of gonotrophic cycles in these females [63,64] to provide additional information about the risk for arbovirus transmission in an area. Populations containing a large percentage of old female mosquitoes, such as females that have passed through several gonotrophic cycles, increase the risk for transmission if virus is present in the area.

Florida’s arbovirus risk prediction surveillance system for *Culex*-borne arboviruses has provided information of possible high-risk areas where mosquito control and public health action is required. Equally important, the surveillance information has also been used in deciding there was little transmission risk in the face of an isolated transmission event to a human or animal. Hence efforts to mitigate transmission can be targeted to areas where there are several types of information indicating the risk for substantial transmission. This provides greater accuracy enabling mosquito control to be more efficient, effective, and environmentally proper. The 2005 West Nile epidemic in Pinellas County illustrates the benefit of Florida’s surveillance programs. In 2005, Pinellas County Mosquito Control District (PCMCD) effectively applied adulticides and control efforts in the area of the county circumscribed by three sentinel chicken flocks that had significantly higher WNV transmission compared to their other sentinel flocks. This was in advance of the first WNV human cases in this area. There were eventually 18 human WNV cases in 2005 in Pinellas County. Estimates of potential cases based on the transmission rate to Pinellas County sentinel chickens was 2–20 cases per week. Transmission never expanded outside the identified transmission zone, and transmission ended in early August 2005 well before the usual end of the mosquito season in this area [55]. Surveillance and aggressive vector control helped to reduce the magnitude of the epidemic. The savings were apparent in confining adulticiding and other efforts to the high-risk zone without county-wide spraying. Efficient, effective, and environmentally proper mosquito control was employed.

## 6. Challenges and Conclusions

The benefits from research on mosquito control are many. The accomplishments reviewed above are selected examples. Mosquito control and mosquito-borne disease control will continue to improve with more research. Research is needed to improve RIM, pesticide delivery systems, and arbovirus surveillance in general. New tools are needed to improve mosquito and mosquito-borne disease control. This means new pesticides, new effective biological control strategies, including the novel use of genetically modified organisms, which must be shown to be environmentally safe. Omitted from this review are the challenges in combatting other arboviruses, better prediction for high-risk areas for epidemics, emerging arboviruses, the impact of environmental factors like global climate change, and the establishment of arboviruses in new areas and environments. The challenges to obtaining the required information are reviewed elsewhere [65,66,67,68,69].

Mosquito control throughout the world faces great challenges [70]. For example, the ability of Florida and the U.S. to be effective against *Aedes*-borne arboviruses like dengue virus (DENV), chikungunya virus (CHIKV), yellow fever virus (YFV), and Zika virus (ZIKV) presents new challenges. These arboviruses and their *Aedes* vectors provide different vector-virus systems compared to Florida’s experience with the *Culex*-arbovirus systems like SLEV and WNV. There is a substantial amount of information concerning mosquito control efforts against *Ae. aegypti* in particular that shows the difficulties in providing mosquito control against this species [71,72,73,74,75]. Currently U.S. mosquito control generally does not have much experience with an *Ae. aegypti*-borne virus epidemic. It will be vital for the U.S. to provide sufficient support for research on various arbovirus systems with the goal to improve control capabilities to meet these new challenges and also to meet the challenges for yet unidentified, newly evolving arbovirus threats to human health and well-being.

## Figures and Tables

**Table 1:** Peer reviewed publications from Florida Medical Entomology Laboratory (FMEL) and Public Health and Entomology Research and Education Center (PHEREC) since 1960 on selected Florida mosquitoes, arboviruses, and related subjects with the total number of articles produced on these subjects (Data from PubMed as of 20 July 2016).

Topic	Total Publications Since 1960	PHEREC	FMEL	% of Total by FMEL and PHEREC
*Culex nigripalpus*	197	2	65	34
Florida mosquito ecology	77	1	26	35
Florida mosquito control	392	42	73	29
Florida St. Louis encephalitis	90	1	42	48
Florida West Nile	116	1	37	33
Florida *Aedes aegypti*	232	2	83	37
Florida mosquito pesticides	141	30	15	32
Florida *Aedes*	436	20	123	33
Florida *Culex*	235	26	109	57
